# Castor Oil in Bowel Preparation Regimens for Colon Capsule Endoscopy: A Systematic Review with Meta-Analysis

**DOI:** 10.3390/diagnostics12112795

**Published:** 2022-11-15

**Authors:** Ulrik Deding, Sofie Sajan Jensen, Benedicte Schelde-Olesen, Lasse Kaalby, Thomas Bjørsum-Meyer, Anastasios Koulaouzidis

**Affiliations:** 1 Department of Clinical Research, University of Southern Denmark, 5230 Odense, Denmark; 2 Department of Surgery, Odense University Hospital, 5000 Odense, Denmark; 3 Department of Social Medicine and Public Health, Pomeranian Medical University, 70-204 Szczecin, Poland

**Keywords:** colon capsule endoscopy, castor oil, bowel preparation, bowel cleansing, excretion rate, completion rate, colon cancer, polyps, colorectal cancer, meta-analysis

## Abstract

Completing colon capsule endoscopy (CCE) investigations rely on successful transit and acceptable bowel preparation quality. We investigated the effect of adding castor oil to the CCE bowel preparation regimen on the completion rate using a meta-analysis of existing literature. We conducted a systematic literature search in PubMed, Web of Science, and Embase. Included studies underwent quality assessment, and data for meta-analysis were extracted. Pooled estimates for excretion rate and acceptable bowel preparation rate were calculated. We identified 72 studies matching our search criteria, and six were included in the meta-analysis. Three of the studies had control groups, although two used historical cohorts. The pooled excretion rate (92%) was significantly higher in patients who received castor oil than in those who did not (73%). No significant difference in acceptable colonic cleanliness was observed. Castor oil has been used in a few studies as a booster for CCE. This meta-analysis shows the potential for this medication to improve excretion rates, and castor oil could be actively considered in conjunction with other emerging laxative regimens in CCE. Still, prospective randomized trials with appropriate control groups should be conducted before any conclusions can be drawn. Prospero ID: CRD42022338939.

## 1. Introduction

In recent years, colon capsule endoscopy (CCE) has been employed at an accelerated pace. This is because of the significant impact of the COVID-19 stalemate on the function of endoscopy units worldwide and the UK’s efforts to introduce CCE as a regular clinical service [1]. However, concerns remain about the use of a procedure considered by many that have yet to be honed to the level of a diagnostic colonoscopy. However, recent studies have shown that CCE has the same diagnostic accuracy as a conventional colonoscopy and tends to outperform CT colonography, especially in detecting small/flat adenomas and sessile serrated lesions [2,3,4,5]. One barrier to a broader clinical application of CCE is achieving an acceptable completion rate (CR). Most studies fail to reach the standards of conventional colonoscopy in terms of complete examinations and adequate bowel cleanliness [6]. A common cause of incomplete CCE is an unacceptable bowel preparation. Several modifications to the bowel preparation procedure have been tested in our unit to remedy this, such as introducing chewing gum, coffee and new medication [7,8,9].

Despite extensive scientific efforts, the ‘magic’ regimen that will ensure acceptable CR (or complete colonic transit) remains elusive [10]. Boosters are considered essential in propelling the capsule out of the body. Sodium Phosphate (NaP) boosters have been the most commonly used but have not been associated with higher CRs or adenoma detection rates [10]. Castor oil is a safe laxative derived from the castor bean that is widely available and commonly used in the general population [11,12]. Adding castor oil to a polyethylene glycol (PEG)-based bowel preparation regimen before colonoscopy has decreased the required volume of bowel preparation solution [13]. In terms of rinsing and suction, interventions are not possible during CCE as opposed to colonoscopy; hence, an extensive bowel cleansing with large volumes of fluids is currently recommended before CCE to increase the likelihood of sufficient cleansing quality and, thereby, a complete investigation. In recent years, several clinical trials have investigated the efficacy of castor oil as a booster for CCE [14,15].

In this systematic review with meta-analysis, we aimed to investigate the efficacy of castor oil in patients subjected to CCE on the rates of complete transit and adequate bowel cleanliness and the diagnostic yield (DY). 

## 2. Methods

The systematic review followed the PRISMA guidelines and was registered with PROSPERO (Prospero ID: CRD42022338939).

### 2.1. Search Strategy

A systematic literature search was conducted in the PubMed, Web of Science and Embase databases. We defined three groups of search terms: investigation, comparator and outcome. These three groups each included all relevant terms within the group combined using the Boolean expression “OR” and the groups were then combined in the final search using the Boolean expression “AND”. The ‘*investigation*’ group was used to identify studies on CCE. The ‘*comparator*’ group was used to limit results to references in which castor oil was included in the bowel preparation regimen. Finally, the ‘*outcome*’ group was to restrict results to studies reporting CR and/or DY. Free text search terms with truncation were included, and indexed search terms were identified in the databases’ thesauruses. No limitation for publication year was applied. The final literature search was conducted on 10 June 2022. Specific search strings are provided in Appendix A, with the search strategy in Table A1.

### 2.2. Inclusion and Exclusion Criteria

We decided to include any identified randomized controlled trials, cohorts and cross-sectional studies describing an adult population (≥18 years) where individuals have undergone CCE using castor oil in the bowel preparation regimen. Only articles written in Danish, English, French, Spanish, or German were included. Reviews, conference papers, and case reports were excluded.

### 2.3. Screening of References

Articles, including their abstracts, were exported from each database and imported to Endnote™, version X9 [16]. Duplicates were excluded. The title and abstract of the remaining articles were screened independently by two authors (U.D. and S.S.J.). Papers meeting the inclusion criteria were included for full-text screening. A paper was included for further review in case of discrepancies between screeners. The full-text manuscripts of the included articles were then retrieved and read in detail independently by two authors (U.D. and S.S.J.), who determined which papers did not meet the inclusion criteria. In case of discrepancies, the authors would re-read the article and discuss it again. To ensure that the search was exhaustive, a snowballing principle was applied to screen the reference lists of included studies for references of possible relevance. Reviewers were not blinded to the authors and institutions of the reviewed manuscripts.

### 2.4. Data Handling

From each included study, two individuals (U.D. and S.S.J.) independently extracted data needed for statistical analysis and evaluated the quality using the MINORS index [17] (Table 1). Differing interpretations were solved in the same manner as with discrepancies regarding the inclusion of studies. 

CRs were then calculated as proportions of investigations in the studies (I) which were complete (II), excreted (III), and with acceptable bowel preparation (IV). According to the Leighton-Rex scale, acceptable bowel preparation was defined as good or excellent. Excretion was determined as the excretion of the capsule within the battery lifetime or visualization of the hemorrhoidal plexus. Test completion was defined as acceptable bowel preparation and excretion of the capsule. DYs were calculated as proportions of individuals (I) who had at least one polyp, (II) one polyp >5 mm, or (III) one polyp > 9 mm (polyp detection rate (PDR)), respectively. The polyp sizes reported in the studies were assumed to follow the standard of reporting the largest diameter of the lesion. 

Additional descriptive data were extracted for subgroup analyses. However, these analyses could not be performed due to the insufficient number of articles within each stratum. For this reason, only some of these data were used for descriptive reasons. These data included first author, publication year, data origin (country), year of data collection, study type, type of capsule, indications for CCE, single- or multi-center study, reported bowel/procedure preparation medicine (including boosters and contrast agents), type of reference standard, gender distribution and mean age.

### 2.5. Pooled Statistical Analysis

We calculated the proportions of complete CCE, excreted capsules and proportions with acceptable bowel preparation. The significance level was set at 5%, and 95% confidence intervals (CI) were calculated. All pooled estimates were calculated from the patient data of included studies in random effects models using Freeman-Tukey double arcsine transformation. Further, the pooled odds ratio (OR) for incomplete CCE transit was calculated for studies reporting control group data.

To test the consistency of the results, I^2^ statistics were performed and evaluated by applying thresholds provided by the Cochrane Handbook [18]. However, if fewer than 10 studies were included, they were omitted [19]. Publication bias and small-study effects were investigated using Egger’s test [20] and illustrated by funnel plots. All results from included studies were extracted and compiled in spreadsheets. All data analyses were conducted in Stata 16 [21] using the Metaprop command [22].

## 3. Results

**Figure 1:**
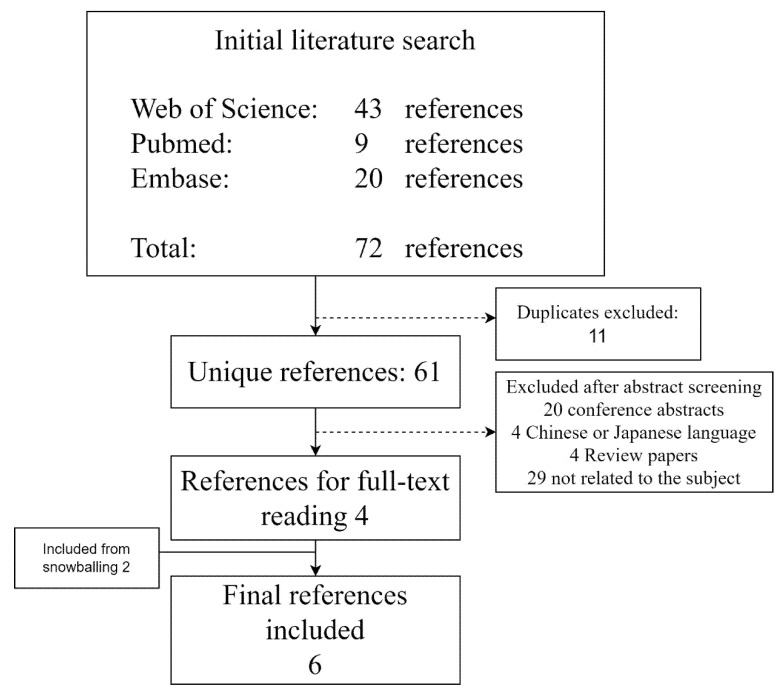
The flow of references from the initial literature search on 10 June 2022 on colon capsule endoscopy using castor oil in the bowel preparation regimen.

The initial literature search resulted in 72 references. Duplicates were removed (11 articles), and 57 articles were excluded after the title and abstract screening. After full-text reading of four papers, they were all included in the study. Thorough snowballing yielded two articles for screening abstracts, of which both were considered eligible for full-text reading and were finally included in the study (Figure 1). In total, six articles were included in this meta-analysis [12,14,15,23,24,25].

An overview of the six included studies is provided in Table 2. Two multi-center and four single-center studies were published between 2016 and 2021; two retrospective, two prospective and two prospective intervention studies with retrospective control groups were identified. All studies used the PillCam^®^ Colon (Medtronic, Minneapolis, MN, USA), although two did not specify the generation; however, as the second generation PillCam^®^ Colon was launched in 2010, it is reasonable to assume they used the second-generation capsule. Five studies were from Japan, and one was from Ireland. The castor oil dose varied from 15 to 90 mL; in all studies, the castor oil was administered as a booster after the ingestion of the capsule. In all studies, PEG-based laxatives were used for bowel preparation before capsule ingestion; in two studies, the split dose regimen was employed. Prokinetics were used in all six studies; in four metoclopramide [12,14,15,24], in one mosapride citrate [25], and in another, a combination of metoclopramide and mosapride citrate as well as suppository [23]. The details of the bowel preparation regimens are presented in Table 3. 

In total, 337 individuals underwent CCE with castor oil in the bowel preparation regimen, and 364 underwent standard CCE without using castor oil in the six included studies. Male participants ranged from 30% to 75%, and the median age ranged from 40 to 67 years. The populations varied between studies and included screening patients [14,25], patients with symptoms [12,14,15,25], ulcerative colitis [24], patients for colonic surveillance [12,23], or after incomplete colonoscopy [12,15], as well as dialysis and kidney transplant patients [23]. None of the studies reported significant adverse events, but in one study [24], two individuals with minor events (nausea and discomfort) were noted. The MINORS index score ranged from 50% of the maximum score to 87.5% (Table 2).

### 3.1. Completion Rate

**Figure 2:**
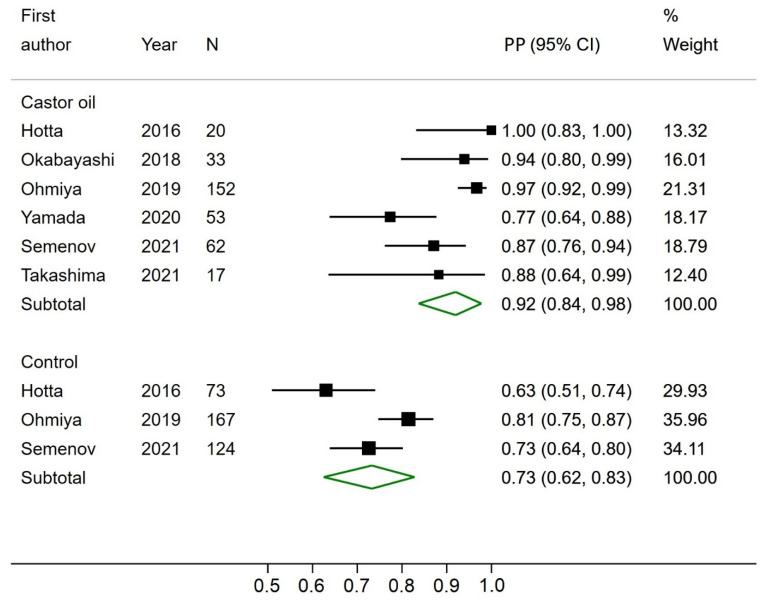
Forest plot of excretion rates of colon capsule endoscopy using castor oil in the bowel preparation regimen and in the control groups. PP—Estimated prevalence proportion.

**Figure 3:**
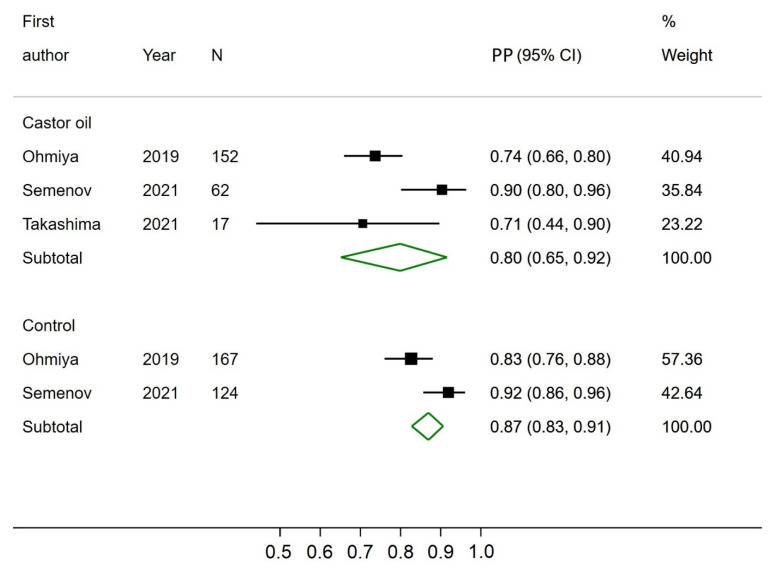
Forest plot of acceptable bowel preparation rates of colon capsule endoscopy using castor oil in the bowel preparation regimen and in the control groups. PP—Estimated prevalence proportion.

None of the six studies reported a CR as defined earlier in this paper. All studies reported the excretion rate (often described as completion), and three studies reported an acceptable bowel preparation rate. Two studies reported the acceptable bowel preparation rate per segment and not overall and could therefore not be included for pooled analysis. Three studies had a control group, of which all reported the excretion rate, and two reported an acceptable bowel preparation rate. The pooled prevalence proportion (PP) of excreted capsules was significantly higher at 92% (CI 95%; 84–98) in CCE using castor oil compared to 73% (CI 95%; 62–83) in the control groups (Figure 2). The pooled PP of acceptable bowel preparation was not significantly different between CCE using castor oil (80%, CI 95%; 65–92) and the control groups (87%, CI 95%; 83–91) (Figure 3). The three studies reporting the proportion of individuals with acceptable bowel preparation used the Leighton–Rex scale [14], the Boston Bowel Preparation Scale [12], and the Aronchick Global Assessment Scale [15], respectively. The two studies reporting bowel preparation per segment had acceptable bowel preparation in 44% and 86% of investigated segments [24,25]. For the three papers reporting control groups, the odds of incomplete CCE transit were significantly reduced in the castor oil group compared to the control groups (OR 0.17, CI95% 0.09; 0.32).

### 3.2. Diagnostic Yield

Two studies reported the PDR, one of which also had a control group for comparison. Therefore, no pooled estimates for DY were calculated. The PDR of CCE with castor oil was 82% in both studies and 44% in the only control group [12,15].

### 3.3. Small Study Effects and Publication Bias

**Figure A1:**
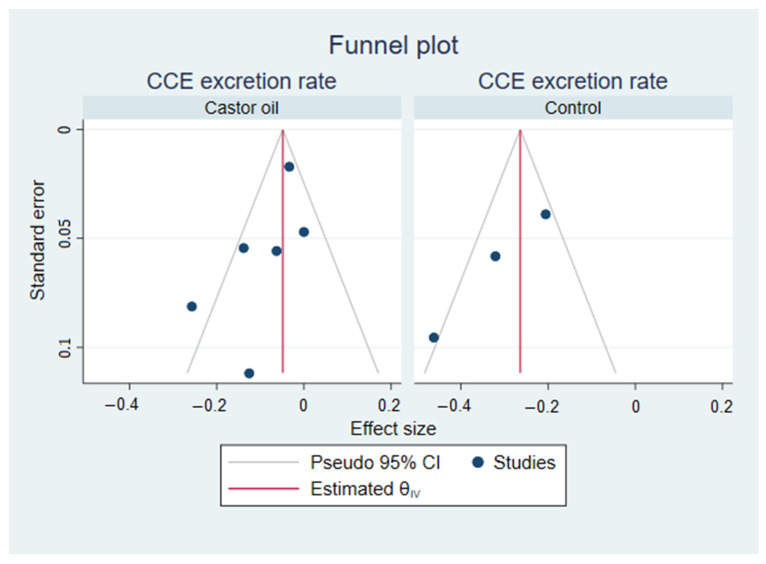
Funnel plots from Egger’s tests visualizing the symmetry of the effect sizes within each sub-analysis of excretion rates from meta-analysis on colon capsule endoscopy using castor oil in the bowel preparation regimen.

**Figure A2:**
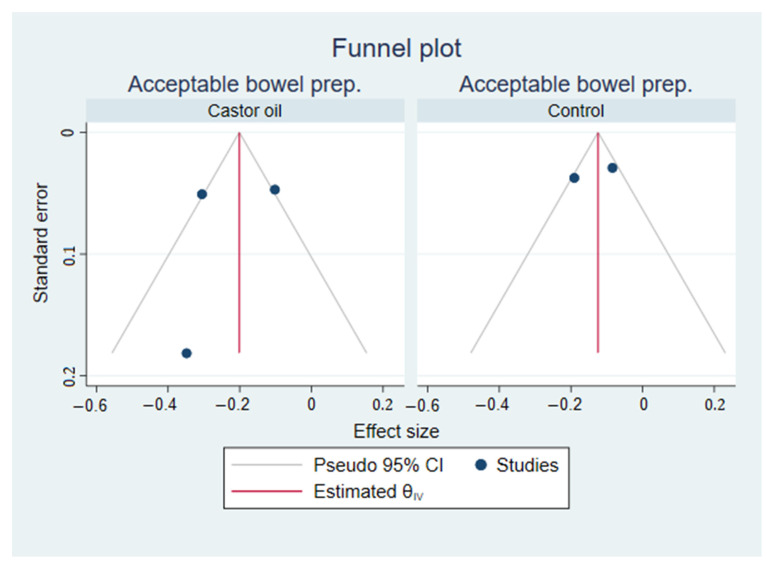
Funnel plots from Egger’s tests visualizing the symmetry of the effect sizes within each sub-analysis of acceptable bowel preparation rate from a meta-analysis on colon capsule endoscopy using castor oil in the bowel preparation regimen.

For small-study effects and publication bias, Egger’s test was performed for each subgroup. Egger’s test was significant (*p* = 0.01) for the CCE excretion rate in control groups but not the castor oil groups (*p* = 0.08). Egger’s test was not significant (*p* = 0.53) for the CCE bowel preparation rate in the castor oil groups and was not conducted in the control groups, as there were only two. Funnel plots are included in Appendix B, Figure A1 and Figure A2.

## 4. Discussion

In the present review, we evaluated the effect of using a castor oil booster on the CR of CCE. The main findings were (a) the proportion of excreted capsules was significantly higher for patients treated with castor oil compared to patients not receiving castor oil, 92% vs. 73%, respectively, and (b) no statistically significant difference was found in the pooled prevalence of acceptable bowel preparation between the castor oil group and the control groups. The PDR was 82% compared to 44% in controls, but this was only reported in one study. Castor oil stimulates peristalsis when hydrolyzed in the small bowel into ricinoleic acid [11,15,26,27]. The odds of incomplete CCE were significantly lower in the castor oil groups compared to the control groups. Caution should be exercised when interpreting the OR, as only one of the included papers included a time-true control group. As castor oil was administered after capsule ingestion as a booster in all the included studies, it seems plausible that this effect on peristalsis would only affect the excretion rate while not improving the cleansing grade achieved.

The bowel preparation regimen in CCE is generally more extensive than that of colonoscopy, as the use of in situ remedying measures for poor bowel preparation, such as rinsing and suction, are not available for CCE. Hence, many studies have investigated different preparation regimens for CCE to increase the rate of excreted capsules and adequate bowel cleanliness [10]. A meta-analysis of 46 studies comprising over 5000 patients reported an overall CR of 0.80 and an acceptable cleanliness rate of 0.77. The highest CR of 0.93 was observed in patients receiving NaP + Gastrografin boosters. The excretion rate from our current review reached this level among the patients receiving castor oil. The booster is essential in CCE as it propagates the capsule and increases the odds of capsule excretion within the capsule battery lifetime. However, the impact of boosters on capsule forward movement in the GI tract must be balanced against the risk of missing significant pathology due to expeditious colonic transit time. To date, no optimal CCE transit time has been established for diagnostics accuracy, but it is of considerable importance as guidance for future booster trials.

In a recent prospective trial, 2 mg of prucalopride as a booster was found to increase the CR and rate of adequate bowel cleanliness in a bowel cancer screening population [8]. This indicates that the booster may significantly impact bowel cleanliness besides adding to the capsule progression. In the same study, patients in the prucalopride group reported more minor adverse events regarding headache, nausea, fatigue and diarrhea. These findings emphasize that several aspects must be considered to choose the optimal booster. It must be effective in capsule propulsion and tolerable with minimal side effects to maintain patient compliance and avoid reluctance towards CCE. Castor oil is an established over-the-counter drug for treating constipation with a good safety profile and only a limited number of side effects, though it should be avoided during pregnancies, and overdose is possible. Castor oil may therefore be a reasonable low-risk addition to bowel preparation regimens, but the rate of major and minor adverse events should be monitored. 

This review was limited by a low number of studies (*n* = 6). Also, the heterogeneity of the included studies regarding the dose of castor oil and the composition of the bowel preparation regimens was a limiting factor. Moreover, none of the included studies were randomized controlled trials, and half of them (*n* = 3) were retrospective. Well-designed RCTs confirm this review’s findings and conducting a multi-arm study with different dosages of castor oil is necessary to clarify the potential. Moreover, the timing of the booster intake concerning capsule ingestion and the combination of more boosters are issues that need to be addressed, as improvement of the CR of CCE is mandatory to enhance the implementation in clinical practice. Using a personalized medicine approach, a personal bowel preparation regimen tailored to the specific individual may be the future, but the empirical evidence for this has yet to be established. 

## 5. Conclusions

Despite its safety profile and easy sourcing, castor oil has been used in only a small number of studies as a booster for CCE. This meta-analysis shows potential for this medication to improve the CR and polyp detection rate, and castor oil use should be actively considered as a viable component for bowel preparation alongside other emerging laxative regimens in CCE.

## Figures and Tables

**Table 1:** Data extracted for statistical analyses.

**Number**	**Description**
I	Number of individuals/investigations included in the study
II	Number of individuals with complete colon capsule endoscopy
III	Number of investigations with excreted capsule within battery lifetime
IV	Number of investigations with acceptable bowel preparation
V	Individuals with polyp(s) (any size polyp, >5 mm polyp and >9 mm polyp)

**Table 2:** Overview of six studies included for meta-analysis colon capsule endoscopy using castor oil in the bowel preparation regimen.

Publication	Study Type	Single- or Multi-Center	CCE Indications	Male %	Age	Castor Oil Sample Size	Control Group Sample Size	MINORS Score ^†^
Hotta, 2016 [23]	Observational, retro-/prospective *	Single	Dialysis and kidney transplant patients, and possibly renal, hepatic and diabetic diseases	75.0	62.7 (mean)	20	73	12/24
Okabayashi, 2018 [24]	Observational, prospective	Single	Ulcerative colitis	48.5	40 (median)	33	-	14/16
Ohmiya, 2019 [14]	Observational, retrospective	Multi	Suspected colorectal diseases: Screening and symptomatic	66.4	58.5 (median)	152	167	17/24
Yamada, 2020 [25]	Observational, retrospective	Multi	FIT positive, screening before surgery, after polypectomy, weight loss, abdominal pain, or diarrhea/constipation	60.4	67 (median)	53	-	10/16
Semenov, 2021 [12]	Observational, retro-/prospective *	Single	Polyp surveillance, lower GI symptoms, incomplete colonoscopy, anemia, or IBS surveillance	45.2	62 (mean)	62	124	17/24
Takashima, 2021 [15]	Observational, prospective	Multi	Suspected colorectal disease and previous incomplete colonoscopy	30	59.5 (mean)	17	-	12/16

* Intervention prospective and control group retrospective. ^†^ Score from 0–16 (0–24 for comparative studies).

**Table 3:** Bowel preparation regimens from included studies as described in the publications.

**Time**	**Hotta, 2016**	**Okabayashi, 2018**	**Ohmiya, 2019**
Day 2			Sennoside: 3 tablets (36 mg)
Day 1	Picosulfate sodium: 1 packet		Magnesium citrate: 50 g
	Sennoside: 2 tablets		Sodium picosulfate: 10 mL, OR Sennoside: 2 tablets (24 mg)
Day 0	Moviprep containing Gascon Drop: 1.5 L	PEG: 500 mL	Moviprep: 500–1000 mL
	Capsule ingestion with Gascon 4 mL	Capsule ingestion with dimethicone	Sodium picosulfate: 10 mL
	Castor oil: 30 mL		Capsule ingestion following Mosapride: 4 tablets (20 mg)
	Intramuscular injection metoclopramide		
Capsule	Mosapride: 4 tablets	PEG: 500 mL	Castor oil: 30 mL
in small bowel	Moviprep: 0.25 L	Castor oil: 20 mL	Moviprep: 1000–1500 mL
		If capsule in stomach at 10 am	Daikenchuto: 5–10 mg (2–4 packs)
		Metoclopramide: 10 mg tablet	If capsule in stomach at 11 am
			Metoclopramide: 10 mg i.m. or i.v. injection
+1 h	Castor oil: 30 mL		
	Teleminsoft suppository		
	Moviprep: 0.25 L		
+1 h	Mosapride: 6 tablets		
	Magcorol P: 1 package		
	*Only dialysis patients:*	If capsule has not been excreted	
	Castor oil: 30 mL	PEG: 500 mL	
	Glycerol enema at 15.30		
+3 h		If capsule has not been excreted	If capsule has not been excreted:
		PEG: 500 mL	Metoclopramide: 10 mg i.m. injection
			Castor oil: 30 mL
			Magnesium citrate: 50 g
**Time**	**Yamada, 2020**	Semenov, 2021 *	**Takashima, 2021**
Day 7		Senna tablets: 4 tablets (48 mg)	
Day 1	Senna tablets: 2 tablets	Moviprep: 1 L	Magnesium citrate P: 50 g
Day 0	PEG: unknown volume U.V.)	Moviprep: 1 L	PEG: 1000 mL
	Capsule ingestion with mosapride: 20 mg	Capsule ingestion	Capsule ingestion with metoclopramide i.v. 10 mg
+30 min.		Metoclopramide i.v. (optional): 10 mg	
		Erythromycin i.v. (optional): 250 mg	
Capsule	PEG: U.V.	Moviprep: 750 mL	Patient leaves for home
in small bowel	Castor oil: U.V.	Castor oil: 15 mL	When home:
	Sodium picosulfate hydrate; U.V.		Castor oil 20 mL
			PEG: 500 mL
+1 h			PEG: 500 mL
+2 h		Moviprep: 250 mL	Magnesium citrate: 50 g
+2 h			Castor oil: 20 mL
+3 h		Dulcolax suppository (optional): 10 mg	

* Semenov et al. (2021) included water in the reported fluid volumes.

## Data Availability

All data analyzed in this review are available in the published papers included and referenced in this publication.

## Appendix A

*Search strings:*

**PubMed: 9 hits**

(Capsule camera* OR Wireless camera* OR Wireless camera endoscop* OR WCE OR CCE OR Colon capsule endoscop* OR PillCam* OR Pill Cam OR Camera pill* OR Capsule endoscop* OR Endoscop*, Capsule OR Wireless Capsule Endoscop* OR Capsule Endoscop*, Wireless OR Endoscop*, Wireless Capsule OR Video Capsule Endoscop* OR Capsule Endoscop*, Video OR Capsule Endoscopy, Video OR Endoscop*, Video Capsule) AND (Castor oil OR Castor OR Oil) AND (Complet* OR Bowel preparation OR Cleans* OR Diagnostic yield OR Transit OR Polyp OR Adenoma OR Detection OR Leighton* OR Vizuali*)

**Web of Science: 43 hits**

((ALL = ((Complet* OR Bowel preparation OR Cleans* OR Diagnostic yield OR Transit OR Polyp OR Adenoma OR Detection OR Leighton* OR Vizuali*))) AND ALL = ((Castor oil OR Castor OR Oil))) AND ALL = ((Capsule camera* OR Wireless camera* OR Wireless camera endoscop* OR WCE OR CCE OR Colon capsule endoscop* OR PillCam* OR Pill Cam* OR Camera pill* OR Capsule endoscop* OR Endoscop*, Capsule OR Wireless Capsule Endoscop* OR Capsule Endoscop*, Wireless OR Endoscop*, Wireless Capsule OR Video Capsule Endoscop* OR Capsule Endoscop*, Video OR Capsule Endoscopy, Video OR Endoscop*, Video Capsule))

**Embase: 20 hits**

(“Capsule camera*” or “Wireless camera*” or “Wireless camera endoscop*” or “WCE” or “CCE” or “Colon capsule endoscop*” or “PillCam*” or “Pill Cam*” or “Camera pill*” or “Capsule endoscopy/” or “Capsule endoscope/” or “Capsule endoscop*” or “Endoscop*, Capsule” or “Wireless Capsule Endoscop*” or “Capsule Endoscop*, Wireless” or “Endoscop*, Wireless Capsule” or “Video Capsule Endoscop*” or “Capsule Endoscop*, Video” or “Capsule Endoscopy, Video” or “Endoscop*, Video Capsule”) And (“Castor oil” or “Castor” or “Oil”) And (“Complet*” OR “Bowel preparation” OR “Cleans*” OR “Diagnostic yield” OR “Transit” OR “Polyp” OR “Adenoma” OR “Detection” OR “Leighton*” OR “Vizuali*”)

**Table A1:** Search strategy to identify relevant studies.

	AND		
	Investigation	Indication	Study Design
**OR**	Capsule camera * Wireless camera * Wireless camera endoscop * WCE CCE Colon capsule endoscop * PillCam * Pill Cam * Camera pill* Capsule endoscop* Endoscop *, Capsule Wireless Capsule Endoscop * Capsule Endoscop *, Wireless Endoscop *, Wireless Capsule Video Capsule Endoscop * Capsule Endoscop *, Video Capsule Endoscopy, Video Endoscop *, Video Capsule	Castor oil Castor Oil	Complet * Bowel preparation Cleans * Diagnostic yield Transit Polyp Adenoma Detection Leighton * Vizuali *

*—truncation identifying all terms regardless of term form.
